# Supplementary material for: The MYB-related transcription factor MYPOP acts as a selective regulator of cancer cell growth
Source: Commun Biol. 2026 May 19;9:678. doi: 10.1038/s42003-026-10272-2 (PMC13187161; doi:10.1038/s42003-026-10272-2)
Supplement: Supplementary file 1 — Supplementary information [file 42003_2026_10272_MOESM1_ESM.docx]

# Supplementary information

# The MYB-related Transcription Factor MYPOP acts as a Selective Regulator of Cancer Cell Growth

Johannes Strunk^1^, Alena Hüppner^2^, Mahwish Sial^1^, Matthias Plath^1^, Mika B. Sheriff^1^, Sascha Wagner^1^, Kirsten Freitag^1^, Snježana Mikuličić^1^, Tatjana Döring^1^, Tobias Bopp^3,4,5,9^, Matthias Klein^3,9^, Krishnaraj Rajalingam^6^, Gregory Harms^3,6,9,10^, Federico Marini^7,9^, Annekathrin S. Nedwed^7^, Thomas Hankeln^8^, Carina Osterhof^8^, Marc A. Schneider^11,12^, Alina Henrich^2^, Andrea Nubbemeyer^2^, Martin Suchan^2^, Silke Brill², Mario Perkovic², Barbara Schrörs^2^, Sebastian Kreiter^2^, Anne Kölsch^2^, Mustafa Diken^2^, Luise Florin^1,4,9,^*

^1^ Institute for Virology, University Medical Center of the Johannes Gutenberg-University Mainz, Obere Zahlbacher Straße 67, 55131 Mainz, Germany

^2^ TRON - Translational Oncology at the University Medical Center of the Johannes Gutenberg University Mainz gGmbH, 55131 Mainz, Germany

^3^ Institute of Immunology, University Medical Center of the Johannes Gutenberg-University Mainz, 55131 Mainz, Germany

^4^ University Cancer Center Mainz, University Medical Center of the Johannes Gutenberg-University Mainz, 55131 Mainz, Germany

^5^ German Cancer Consortium (DKTK), 69120 Heidelberg, Germany

^6^ Cell Biology Unit, University Medical Center of the Johannes Gutenberg University Mainz, 55131 Mainz, Germany

^7^ Institute of Medical Biostatistics, Epidemiology and Informatics (IMBEI), University Medical Center of the Johannes Gutenberg University Mainz, Rhabanusstraße 3, 55118 Mainz, Germany.

^8^ Institute of Organismic and Molecular Evolution, Molecular Genetics and Genome Analysis Group, Department of Biology, Johannes Gutenberg University Mainz, J. J. Becher-Weg 30A, 55128 Mainz, Germany

^9^ Research Center for Immunotherapy (FZI), University Medical Center of the Johannes Gutenberg-University Mainz, 55131 Mainz, Germany

^10^ Wilkes University, Department of Biology, 84 W. South Street, Wilkes Barre, PA 18766, USA

^11^ Translational Lung Research Center (TLRC) Heidelberg, German Center for Lung Research (DZL), 69126 Heidelberg, Germany

^12^ Translational Research Unit (STF), Thoraxklinik at Heidelberg University Hospital, 69126 Heidelberg, Germany

**
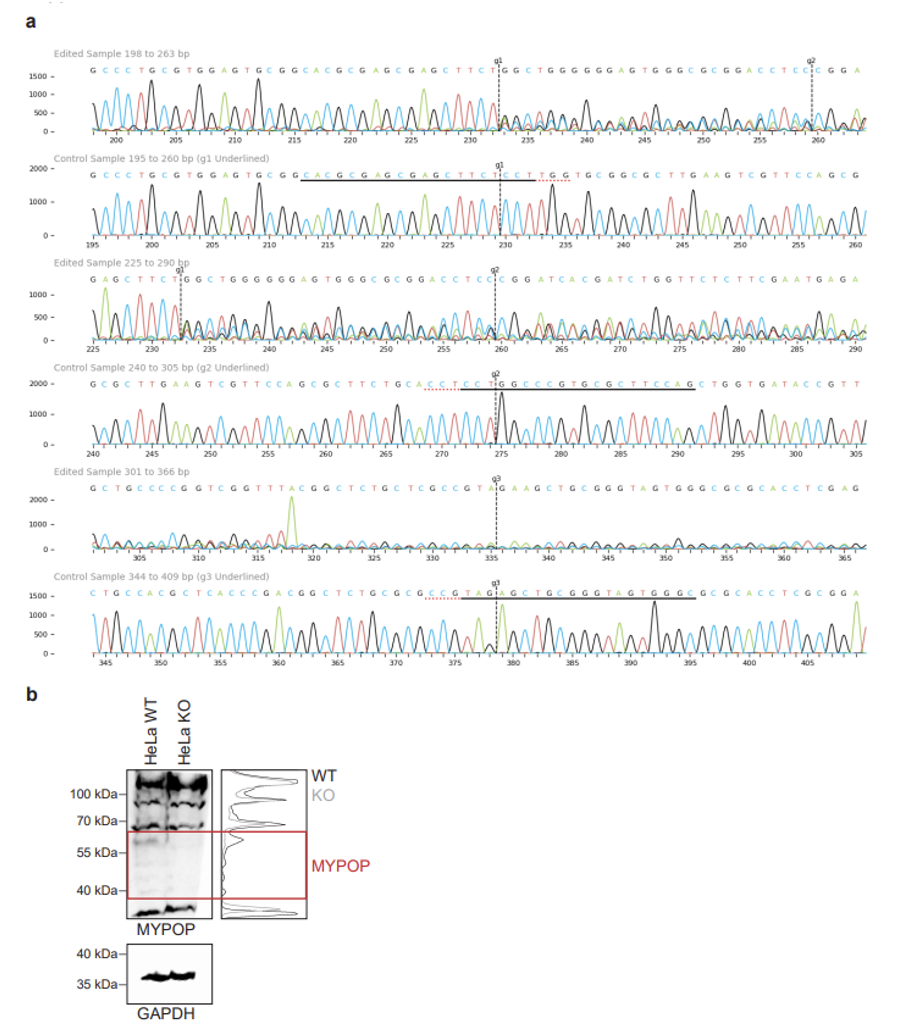
**

**Supplementary Fig. 1: Validation of MYPOP knockout by Sanger sequencing and protein analysis. a)** Sanger sequencing of genomic DNA from wild-type (WT) cells (control sample) shows clean, single-peak traces across the MYPOP locus, whereas MYPOP-KO cells (edited sample) display mixed, overlapping signals beginning at the guide RNA (gRNA; g1–g3) target sites, indicative of indels and confirming successful gene disruption. gRNA sequences are underlined; red and black dashed lines mark predicted Cas9 recognition (Protospacer adjacent Motif) and cleavage sites, respectively. **b)** Western blot analysis of whole-cell lysates from WT and MYPOP-KO HeLa cells using a polyclonal rabbit anti-MYPOP antibody (ab221487, Abcam). Multiple non-specific and specific MYPOP-related bands are detected. Histogram (right) demonstrates a marked reduction of the ~60-kDa band in KO cells, identifying this band in WT lysates as endogenous MYPOP. Additional faint bands between 40–60 kDa may represent less-modified, cleaved, or alternative MYPOP isoforms.

**Supplementary Fig. 2: HPV18 E6 and E7 transcripts are reduced after transfection with GFP-MYPOP.** Relative expression values of HPV18 E6 and E7 genes from RNA-Seq experiments with HeLa cells and overexpressed GFP-MYPOP 24h p.t. Normalized counts (DESeq2, median of ratios) from control treated samples were set to 100% indicated by a dotted line. Statistical significance (n=3) was determined between control and GFP-MYPOP samples with p = 0.1465 for E6 and with p = 0.0802 for E7.


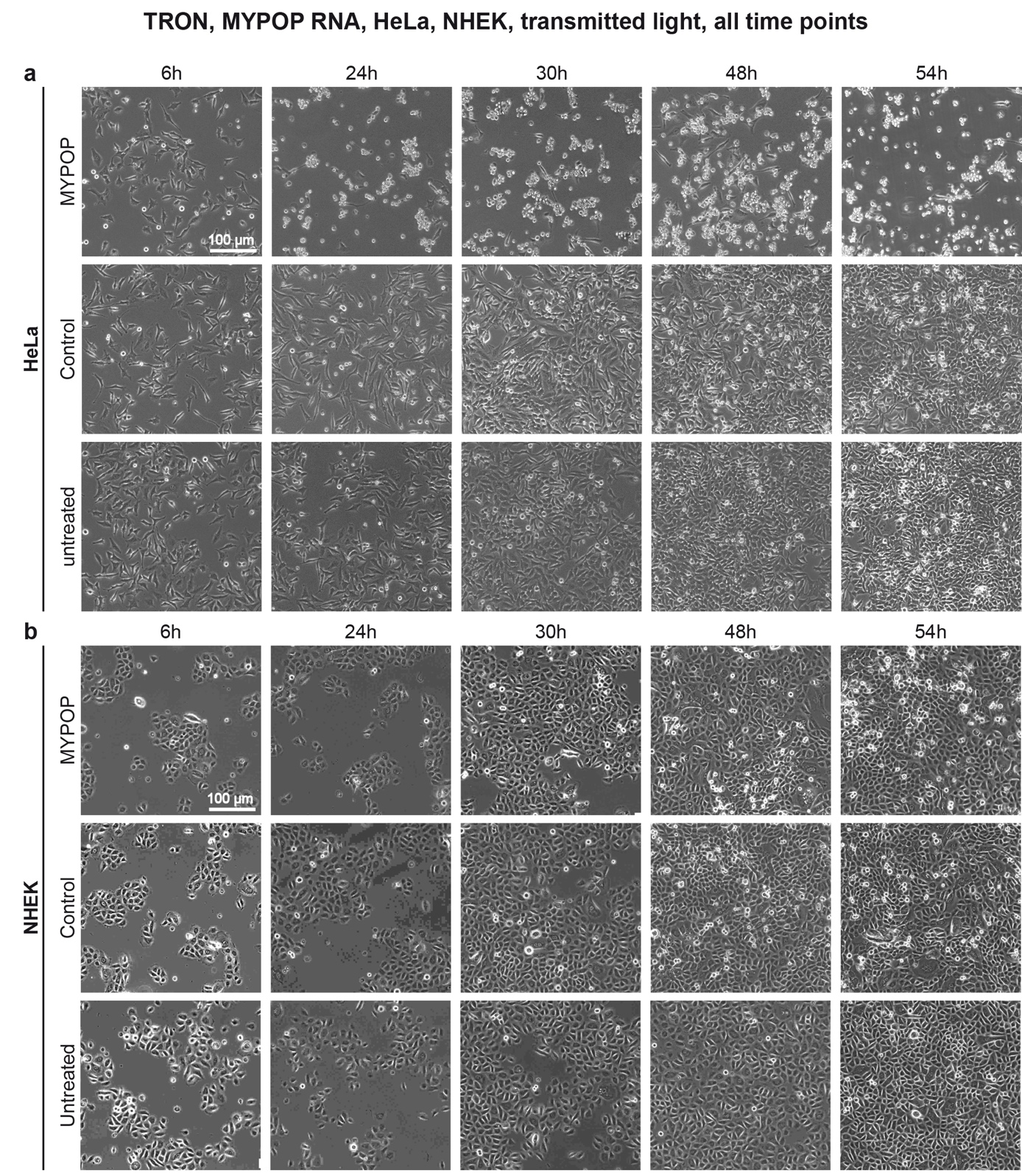


**Supplementary Fig. 3: The effect of MYPOP mRNA transfection on morphology and cell growth of HeLa and NHEK cells.** Optical microscope overview images of untreated, control transfected and MYPOP transfected HeLa cells (**a**) and NHEK cells (**b**) at time points between 6 and 54 hours post mRNA transfection.


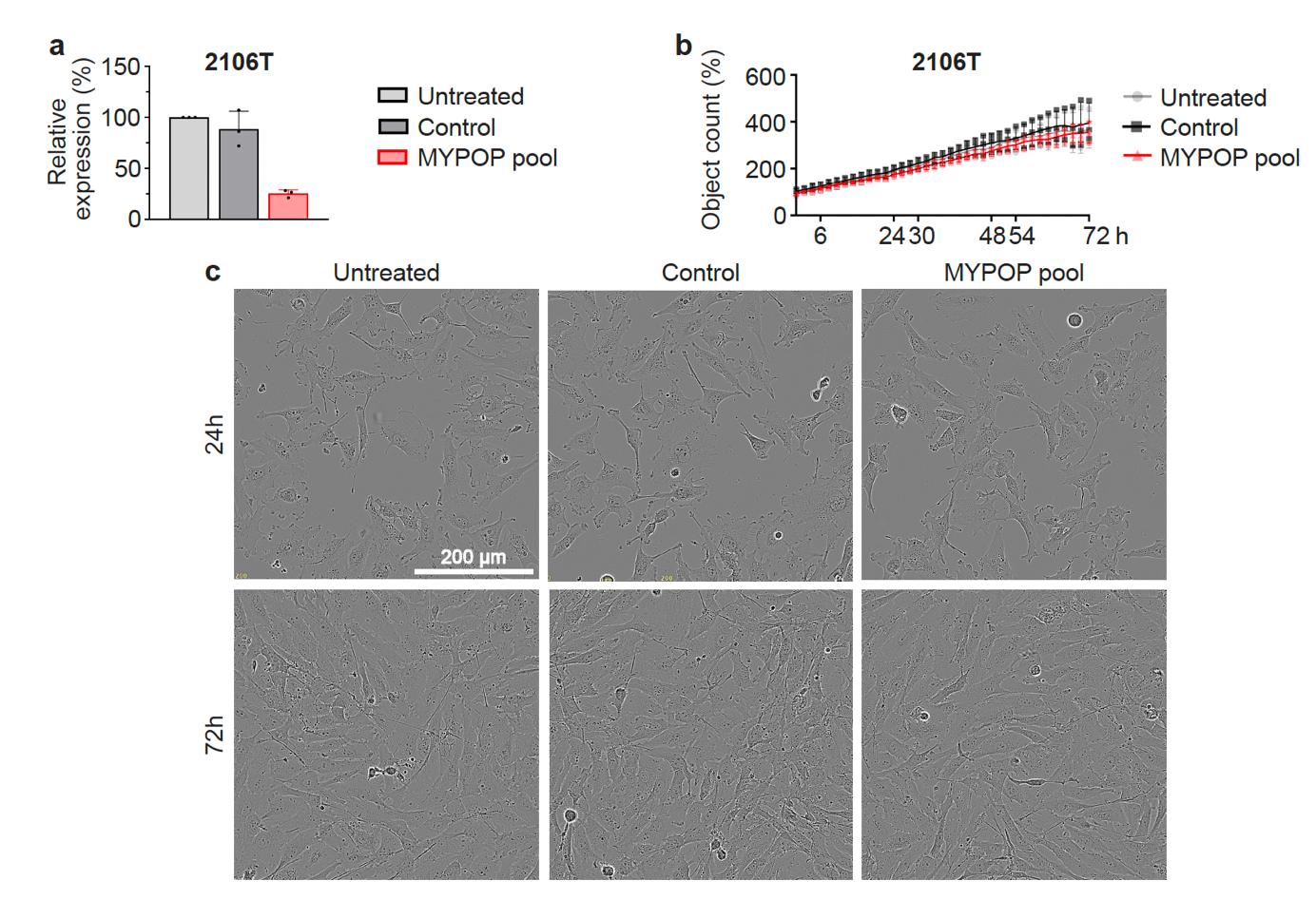


**Supplementary Fig. 4: MYPOP siRNA treatment has no effect on cell growth or cell morphology of 2106T cells.** 40,000 2106T cells per well were seeded in 12-well plate. After 24 hours cells were transfected either with ON-TARGETplus GAPD Control Pool (Horizon # D-001830-10-05) or ON-TARGETplus Human MYPOP (339344) siRNA – SMARTpool (Horizon, #L-023759-02-0020). Per reaction 30 nM siRNA were used. As further control, untreated cells were included. All samples were prepared in triplicates. Transfection was performed using Lipofectamine MessengerMAX mRNA Transfection Reagent (LMRNA, Thermo Fisher Scientific, USA) according to the manufacturer's protocol. **a)** Validation of *MYPOP* by RT-qPCR in 2106T cells after siRNA-mediated depletion. Statistical significance (n=3) was determined between Control and *MYPOP*-pool siRNAs with p = 0.0212. **b)** For visual monitoring, 12-well plate was placed in Incucyte® Live Cell Analysis System. Growth curve of untreated, control siRNA, and MYPOP pool siRNA treated cells at the indicated time points. Statistical significance (n=3) was determined between Control and *MYPOP*-specific siRNA with p = 0.5356 at 72h p.t. **c)** Optical microscope overview images of untreated, control siRNA, and *MYPOP*-pool siRNA treated cells.


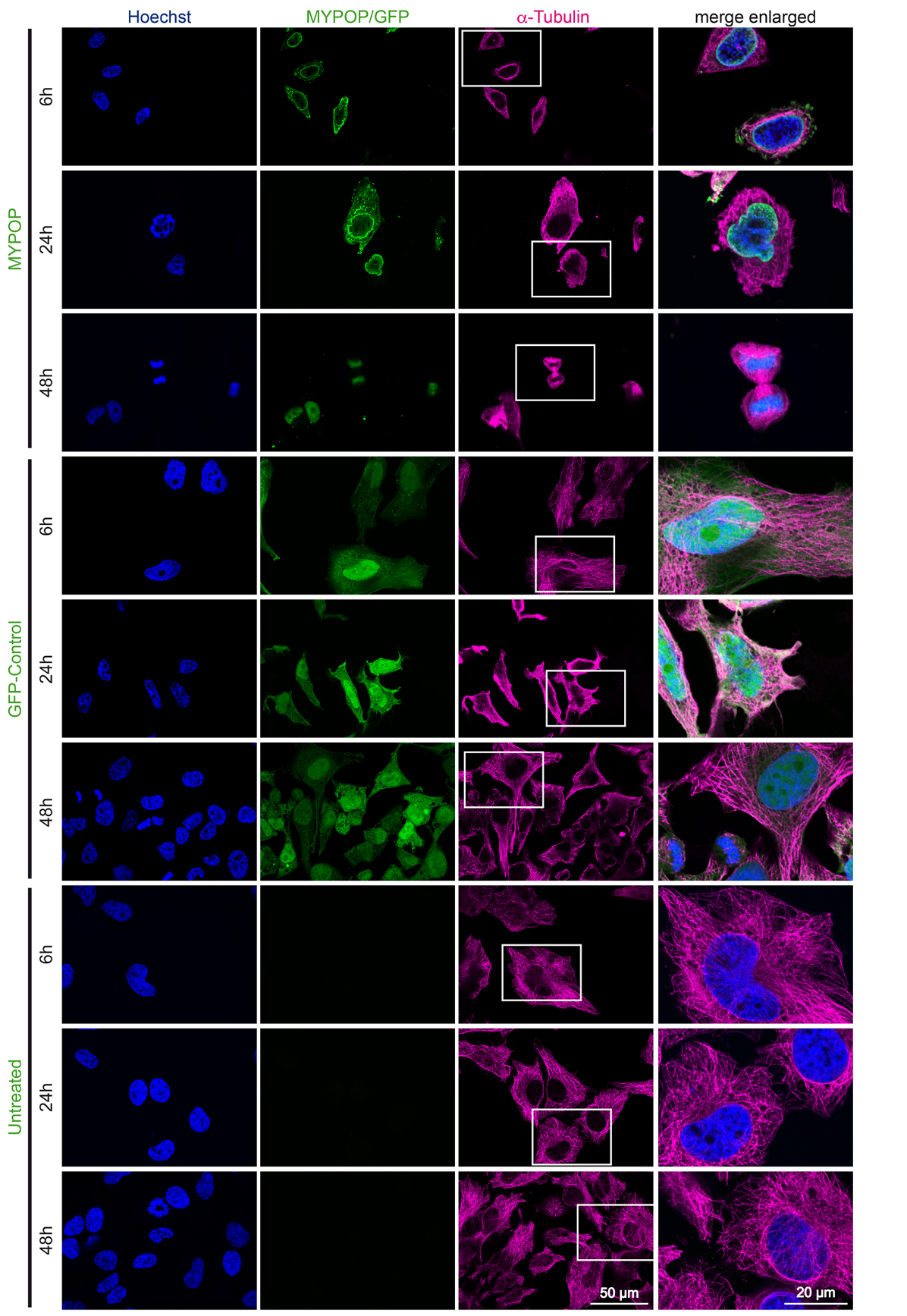


**Supplementary Fig. 5: mRNA transfection efficiency, protein localization, and MYPOP effect on HeLa cells.** Representative confocal laser scanning microscopy images of HeLa cells show transfection efficiency, protein localization and the shrinkage of cells and cell nuclei as well as the effect of MYPOP on the α-tubulin network at 6, 24 and 48 hours post transfection. Staining was performed as in legend to Fig.4 with α-tubulin shown in magenta, MYPOP shown in green and chromatin in blue (Hoechst 33342). A white box in the α-tubulin images indicates the area that was used for the enlarged images.

**
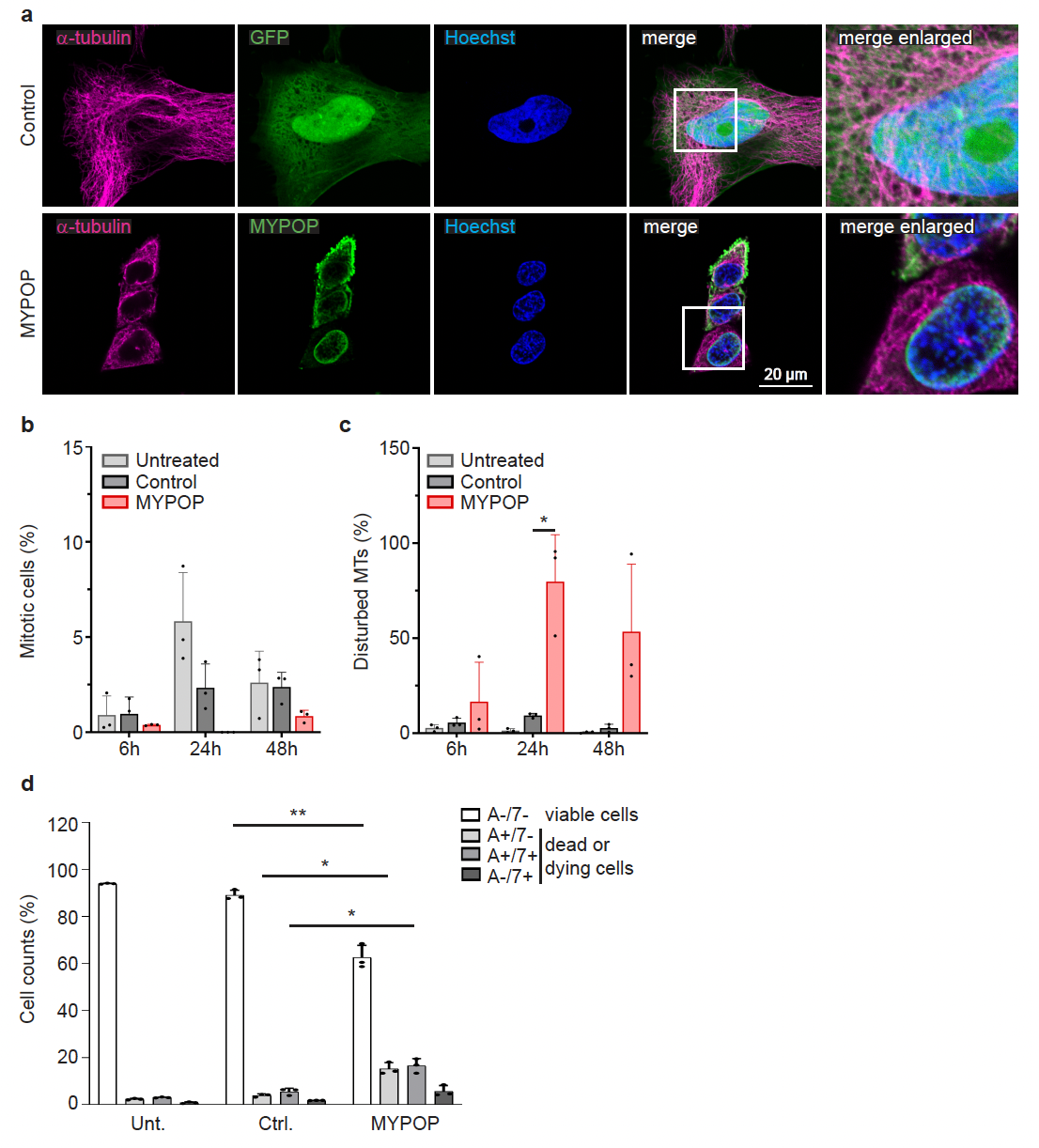
Supplementary Fig. 6: MYPOP expression after mRNA transfection induces microtubule perturbation and prevents mitosis.** **a)** Representative confocal laser scanning microscopy images of HeLa cells show the shrinkage of cells and cell nuclei as well as the effect of MYPOP on the α-tubulin network. Staining was performed as in legend to Fig.4 with α-tubulin shown in magenta, GFP and MYPOP shown in green and chromatin in blue (Hoechst 33342). A white box in the merge indicates the area that is shown in the enlarged images. **b, c)** Cell divisions and disturbed microtubule networks were quantified at 6h, 24h and 48h p.t. comparing untreated control transfected and MYPOP transfected HeLa cells. At least 100 cells were included for each biological replicate. Values (n=3) are shown as mean + SD. Statistical significance was determined comparing Control and MYPOP for mitotic cell quantification with p (6h) = 0.382, p (24h) = 0.084, p (48h) = 0.0593 and for disturbed microtubule networks with p (6h) = 0.4551, p (24h) = 0.0384 and p (48h) = 0.1312. **d)** Quantification of cell death assays which were performed by staining untreated (Unt.), control mRNA transfected (Ctrl.) and MYPOP mRNA transfected (MYP.) cells 24h p.t. with Annexin V-PE and 7-AAD (used as described in legend to Fig. 2). Values (n = 3) are shown as mean + SD. Statistical significance was determined with p (A-/7-) = 0.0058 (displayed in the figure), p (A+/7-) = 0.0122, p (A+/7+) = 0.0126 and p (A-/7+) = 0.1046 comparing Control (Ctrl.) and MYPOP.


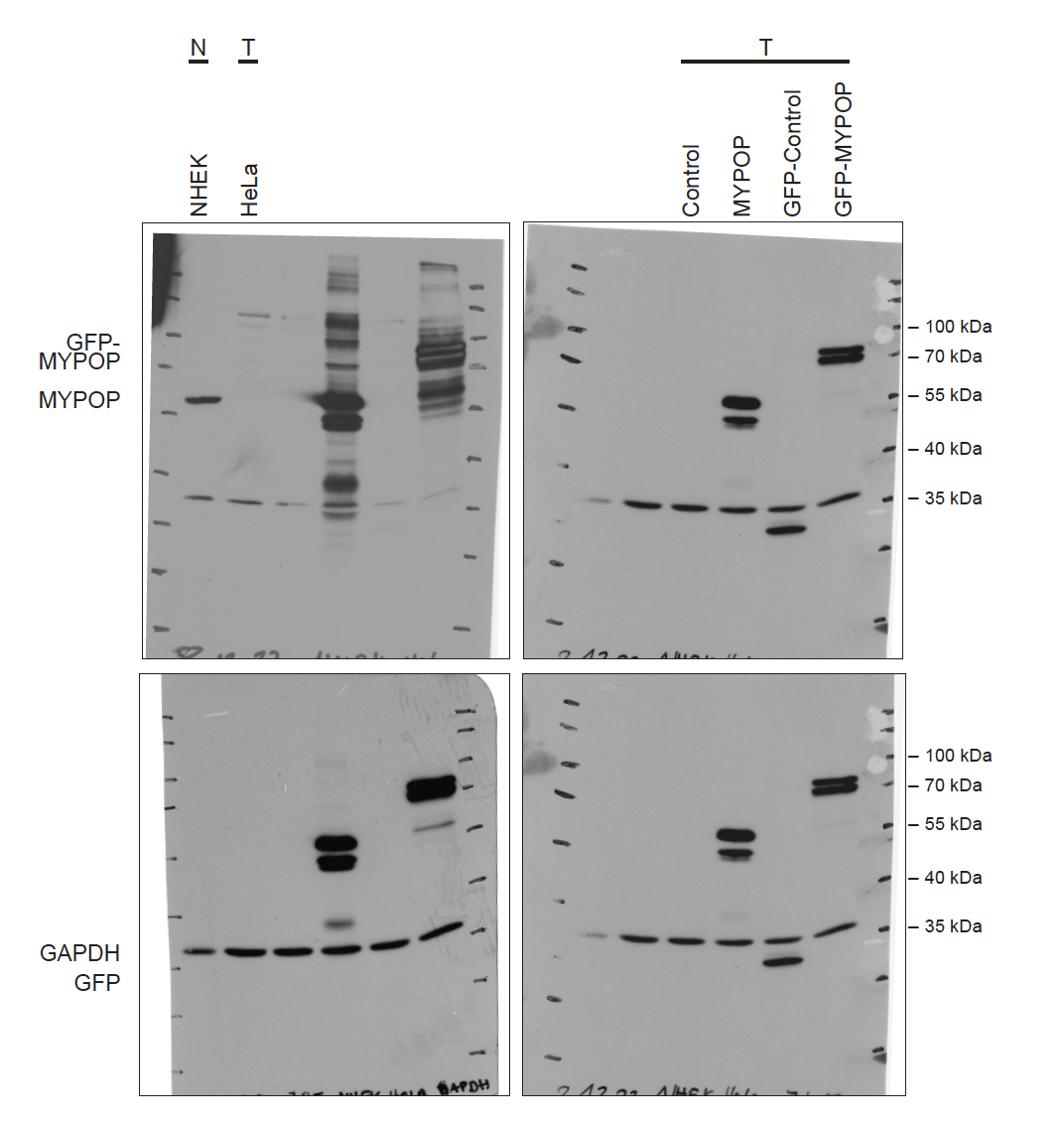


**Supplementary Fig. 7: Uncropped blots shown in Fig. 1a**


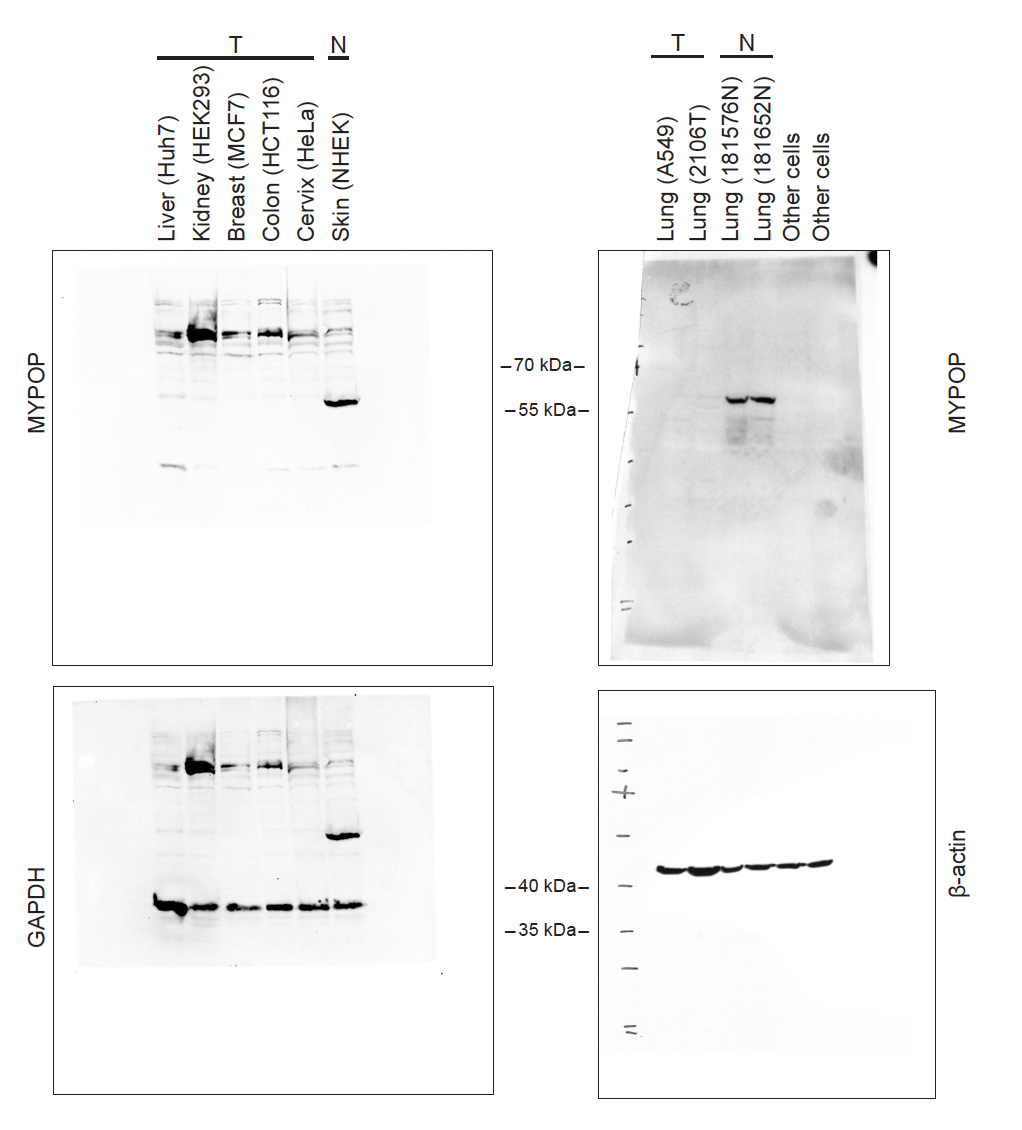
**Supplementary Fig. 8: Uncropped blots shown in Fig. 5a**

**
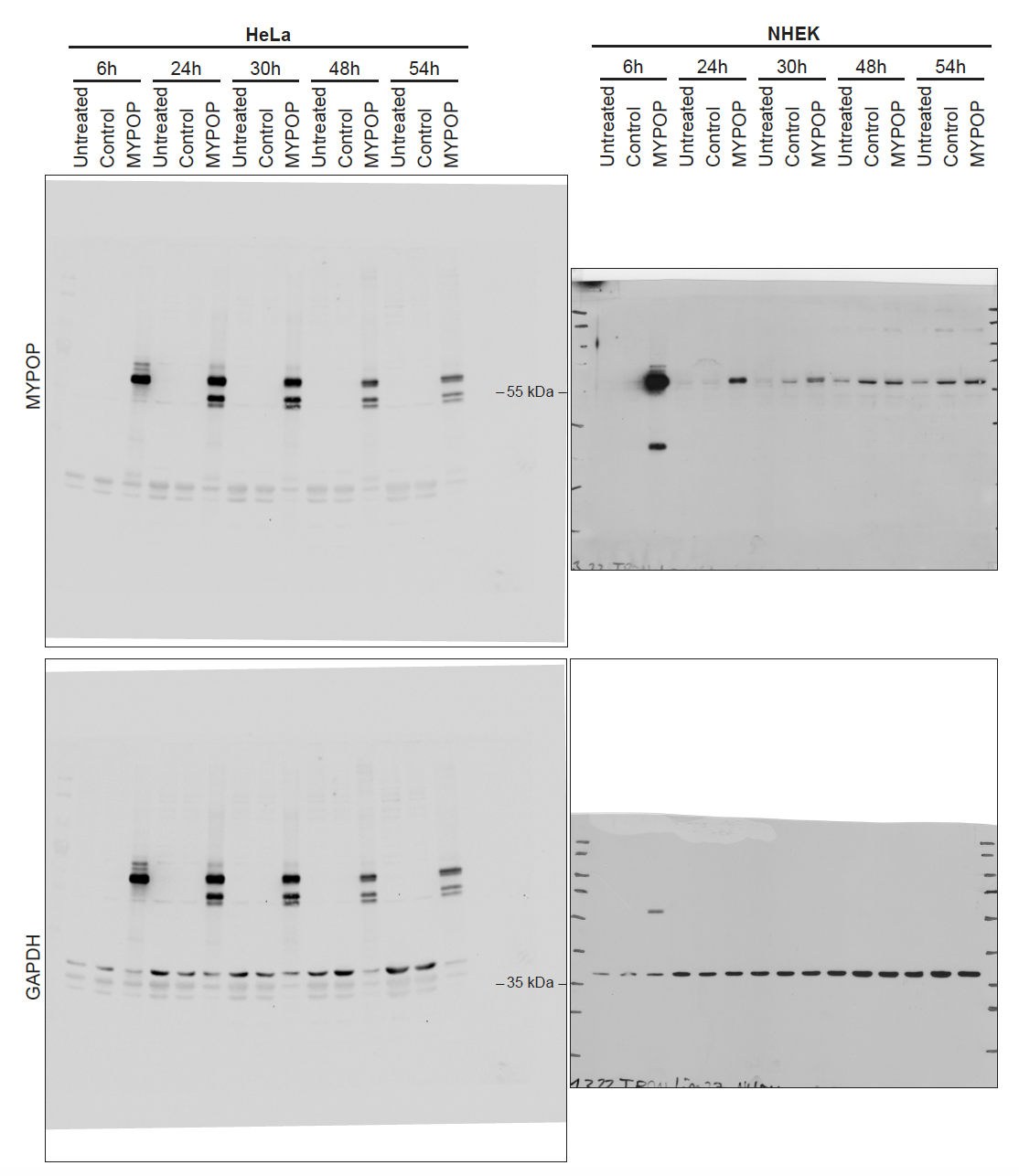
**

**Supplementary Fig. 9: Uncropped blots shown in Fig. 6a**

**Supplementary Fig. 10: Uncropped blots shown in Fig. 6c**

**Supplementary Fig. 11: Uncropped blots shown in Fig. 10a**

**Supplementary Fig. 12: Uncropped blots shown in Fig. Supplementary Fig. 1a**

**Supplementary Fig. 13: Gating strategy for identification of GFP-/GFP-MYPOP positive cells and apoptosis analysis of GFP-/GFP-MYPOP positive HeLa cells.** **a)** HeLa cells expressing GFP (GFP-positive cells) were first identified by flow cytometry based on GFP fluorescence intensity separating the two populations: GFP-positive and GFP-negative cells (FSC versus GFP). GFP-positive cells are displayed as green dots and then displayed as SSC versus FSC. GFP- and GFP-MYPOP-positive cells were selected for downstream analysis. **b)** Apoptosis was then assessed within the GFP-positive cell population using Annexin V-PE and 7-AAD staining analysis. For GFP-positive samples, quadrant gates were established using unstained control cells, with approximately 98% of events positioned within the Annexin V⁻/7-AAD⁻ lower right quadrant. Cutoff lines for Annexin V-PE and 7-AAD were defined using a representative Annexin V-PE and 7-AAD stained sample such that all subpopulations were clearly separated. These gate settings were subsequently applied uniformly across all samples to ensure consistency and comparability.

## Supplementary Table 1: Primer list

| **Gene** | **Sequence 5‘ to 3‘** | **Reference /Accession No.** |
| --- | --- | --- |
| GAPDH | F: GTGAAGGTCGGAGTCAACGG  R: TGACAAGCTTCCCGTTCTCA | NM_002046.7 |
| HPV18 E6 | F: GTGCCAGAAACCGTTGAATCC  R: CGAATGGCACTGGCCTCTAT | NC_001357.1 |
| HPV18 E7 | F: ACATTTACCAGCCCGACGAG  R: GGTCGTCTGCTGAGCTTTCT | NC_001357.1 |
| IL11 | F: GACCACAACCTGGATTCCCT  R: AGGTAGGACAGTAGGTCCGC | NM_000641.4; NM_001267718.2 |
| IL1A | F: CGCCAATGACTCAGAGGAAGA  R: AGGGCGTCATTCAGGATGAA | Nasser et al. 2012^92^ |
| IL20 | F: CATTGTGGGGAGGAAGCAATG  R: TAGTTCCCCCAAAGCCTTCAC | NM_018724.4 |
| IL24 | F: GCTTCTCTGGAGCCAGGTATCA  R: ACATCTCATTTTCTTGCGAGACG | NM_001185157.1 |
| MYC | F: CAGCGACTCTGAGGAGGAAC  R: CCCTCTTGGCAGCAGGATAG | NM_002467.6 |
| MYPOP | F: ACAGAAGGGCCGATACAAGC  R: CTTTAGGTCTAGGGCAGCCG | - |
