# Supplementary material for: The MYB-related transcription factor MYPOP acts as a selective regulator of cancer cell growth
Source: Commun Biol. 2026 May 19;9:678. doi: 10.1038/s42003-026-10272-2 (PMC13187161; doi:10.1038/s42003-026-10272-2)
Supplement: Supplementary file 2 — Description of Additional Supplementary File [file 42003_2026_10272_MOESM2_ESM.docx]

## description of Supplementary Data files_Strunk et al., 2026

## Supplementary Data 1: Processed files for RNA-Seq datasets. RNA-Seq results of HeLa 24h post pDNA, 6h and 24h post mRNA as well as NHEK 6h and 24h post mRNA transfection. All DEGs with an adjusted p-value ≤ 0.05 are listed according to the fold change. Shown are gene symbols, stable identifiers (ENSEMBL ID) as well as log_2_-fold change values, p-values and adjusted p-values for each DEG, respectively.

**Supplementary Data 2: Numerical source data.** Numerical source data for the graphs in the manuscript.
