# Supplementary material for: The MYB-related transcription factor MYPOP acts as a selective regulator of cancer cell growth
Source: Commun Biol. 2026 May 19;9:678. doi: 10.1038/s42003-026-10272-2 (PMC13187161; doi:10.1038/s42003-026-10272-2)
Supplement: Supplementary file 5 — Reporting Summary [file 42003_2026_10272_MOESM5_ESM.pdf]

nature portfolio

Corresponding author(s): Luise Florin

Last updated by author(s): Apr 19, 2026

Reporting Summary

Nature Portfolio wishes to improve the reproducibility of the work that we publish. This form provides structure for consistency and transparency in reporting. For further information on Nature Portfolio policies, see our Editorial Policies and the Editorial Policy Checklist.

Statistics

For all statistical analyses, confirm that the following items are present in the figure legend, table legend, main text, or Methods section.

n/a	Confirmed	
☐	☒	The exact sample size (*n*) for each experimental group/condition, given as a discrete number and unit of measurement
☐	☒	A statement on whether measurements were taken from distinct samples or whether the same sample was measured repeatedly
☐	☒	The statistical test(s) used AND whether they are one- or two-sided<br>*Only common tests should be described solely by name; describe more complex techniques in the Methods section.*
☒	☐	A description of all covariates tested
☐	☒	A description of any assumptions or corrections, such as tests of normality and adjustment for multiple comparisons
☐	☒	A full description of the statistical parameters including central tendency (e.g. means) or other basic estimates (e.g. regression coefficient) AND variation (e.g. standard deviation) or associated estimates of uncertainty (e.g. confidence intervals)
☐	☒	For null hypothesis testing, the test statistic (e.g. *F*, *t*, *r*) with confidence intervals, effect sizes, degrees of freedom and *P* value noted<br>*Give P values as exact values whenever suitable.*
☒	☐	For Bayesian analysis, information on the choice of priors and Markov chain Monte Carlo settings
☒	☐	For hierarchical and complex designs, identification of the appropriate level for tests and full reporting of outcomes
☒	☐	Estimates of effect sizes (e.g. Cohen's *d*, Pearson's *r*), indicating how they were calculated

Our web collection on statistics for biologists contains articles on many of the points above.

Software and code

Policy information about availability of computer code

Data collection	Axiovision 4.7, Carl Zeiss, Germany for collection of immunofluorescence images.<br>LASX Software, Leica Mannheim, Germany used for collection of higher resolution immunofluorescence images.<br>CXP analysis software (Beckman Coulter, USA) for the collection of flow cytometry data.<br>FlowJo v10.8.1 software for the collection of flow cytometry data.
Data analysis	GraphPad Prism 9, version 9.5.0 used for statistical data analysis<br>FlowJo v10.8.1 software used for FC data analysis<br>CXP analysis software (Beckman Coulter, USA) used for FC<br>ELISA data was analysed using GainData® (arigo Biolaboratories Corp., Taiwan).<br>LegendPlex Data was analysed using the QOGNIT LEGENDplex™ Data Analysis Software Suite (version 2023-02-15).<br>RNA seq: Quality control on the sequencing data was performed with the FastQC tool (version 0.11.2, https://www.bioinformatics.babraham.ac.uk/projects/fastqc/). Alignments were quantified with the featureCounts function of the Rsubread package (version 2.10.5) against the ENSEMBL v107 annotation. Exploratory data analysis was performed with the pcaExplorer package (version 2.22.0). For batch effect analysis the limma package (version 3.52.4) and the sva package (version 3.44.0) were used. As result, the batch of the samples was included in the different expression analysis model as well as the condition of the samples. Differential expression analysis was performed with edgeR package (version 3.38.4), setting the false discovery rate (FDR) cutoff to 0.05. KEGG pathway enrichment was performed with the enrichr package (version 3.1) and the KEGG_2019_human database. The enrichment results were further processed with the GeneTonic package for visualization and summarizing (version 2.0.2). Gene expression profiles were plotted as heatmaps (color-coded standardized z-scores for the expression values, after variance stabilizing transformation using the DESeq2 package (version 1.36.0) to simplify comparison across samples.

STRING analysis (version 11.5, https://string-db.org/) was conducted with all DEGs which were included in the KEGG Pathway 'cell cycle'. Minimum required interaction score was set to highest confidence (0.900). Quality control on the sequencing data was performed with the FastQ Screen tool (0.15.1) and the FastQC tool (version 0.11.9). RNA sequencing reads were processed with kallisto (version 0.42.4) and the Homo sapiens reference GRCh38 (ENSEMBL release). Estimated read counts were summarized per gene and used for differential expression analysis with DESeq2 (version 1.34.0). Log2 fold change values after "apeglm" shrinkage 90 are shown. Genes with BH91 adjusted p-values < 0.05 were considered as significantly regulated.
Apart from RNA-Seq experiments, statistical data analysis was performed using GraphPad Prism 9 (version 9.5.0).

For manuscripts utilizing custom algorithms or software that are central to the research but not yet described in published literature, software must be made available to editors and reviewers. We strongly encourage code deposition in a community repository (e.g. GitHub). See the Nature Portfolio guidelines for submitting code & software for further information.

Data

Policy information about availability of data

All manuscripts must include a data availability statement. This statement should provide the following information, where applicable:

- Accession codes, unique identifiers, or web links for publicly available datasets
- A description of any restrictions on data availability
- For clinical datasets or third party data, please ensure that the statement adheres to our policy

Raw and processed files for RNA-Seq datasets generated in the scope of this manuscript using pDNA constructs for transfection are available in the Gene Expression Omnibus (GEO) database under the accession number GSE260896.
The data of the mRNA-mediated MYPOP gene transfer has been deposited in the European Nucleotide Archive (ENA) at EMBL-EBI under accession number PRJEB70469 (https://www.ebi.ac.uk/ena/browser/view/PRJEB70469). Processed files for RNA-Seq datasets are provided in Supplementary Tables 1-5. Numerical source data for all graphs with error bars or statistical analyses from biological replicates are provided in Supplementary Table 7.

Research involving human participants, their data, or biological material

Policy information about studies with human participants or human data. See also policy information about sex, gender (identity/presentation), and sexual orientation and race, ethnicity and racism.

Reporting on sex and gender	This study does not include human participants, their data, or biological material.
Reporting on race, ethnicity, or other socially relevant groupings	This study does not include human participants, their data, or biological material.
Population characteristics	This study does not include human participants, their data, or biological material.
Recruitment	This study does not include human participants, their data, or biological material.
Ethics oversight	This study does not include human participants, their data, or biological material.

Note that full information on the approval of the study protocol must also be provided in the manuscript.

Field-specific reporting

Please select the one below that is the best fit for your research. If you are not sure, read the appropriate sections before making your selection.

☒ Life sciences ☐ Behavioural & social sciences ☐ Ecological, evolutionary & environmental sciences

For a reference copy of the document with all sections, see nature.com/documents/nr-reporting-summary-flat.pdf

Life sciences study design

All studies must disclose on these points even when the disclosure is negative.

Sample size	At least three biological distinct samples were used for each experiment. The minimum of three replicates was chosen to allow statistical analysis.
Data exclusions	No data were excluded from the study.
Replication	The study design ensures for reproducibility. A majority of the results were obtained from two different laboratories using two distinct transfection systems (plasmid DNA vs. mRNA)
Randomization	Cell culture samples were randomly allocated into control or treatment groups. Both groups were otherwise treated under the same conditions.
Blinding	Blinding was conducted during analysis of immunofluorescence microscopy analysis. For other experiments, blinding was not relevant as

Blinding	results were analysed using an unbiased device for data collection and analysis.

Reporting for specific materials, systems and methods

We require information from authors about some types of materials, experimental systems and methods used in many studies. Here, indicate whether each material, system or method listed is relevant to your study. If you are not sure if a list item applies to your research, read the appropriate section before selecting a response.

Materials & experimental systems

n/a	Involved in the study	
☐	☒	Antibodies
☐	☒	Eukaryotic cell lines
☒	☐	Palaeontology and archaeology
☒	☐	Animals and other organisms
☒	☐	Clinical data
☒	☐	Dual use research of concern
☒	☐	Plants

Methods

n/a	Involved in the study	
☒	☐	ChIP-seq
☐	☒	Flow cytometry
☒	☐	MRI-based neuroimaging

Antibodies

Antibodies used	Antibodies used in this study were as follows: anti-MYPOP rabbit polyclonal antibody ab221487 (Abcam, UK); anti-GFP mouse monoclonal antibody (mAb) JL-8 (Clontech, USA); anti-GAPDH mAb 60004-1-Ig (Proteintech, Germany); anti-α-tubulin mAb T5168 (Merck KGaA, Germany); anti-mouse and anti-rabbit horseradish peroxidase-coupled secondary antibodies (Dianova, Germany) Alexa Fluor 488- or Alexa Fluor 546-coupled secondary antibodies (Thermo Fisher Scientific, USA).
Validation	Primary antibodies were validated by the manufacturer (ab221487 - ICC/IF; GAPDH - WB, IP, IF, FC; JL-8 - WB; alpha-tubulin - 'enhanced validation'). Antibodies were also controlled by IF and/or WB assays comparing treated and untreated samples.

Eukaryotic cell lines

Policy information about cell lines and Sex and Gender in Research

Cell line source(s)	HeLa cells were purchased from the German Resource Center of Biological Material (DSMZ, Germany).<br>HEK293 cells were obtained from ATCC (CRL-1573).<br>Murine B16-F10 and CT-26 cells were purchased at ATCC (CRL-6475 and CRL-2638).<br>NHEK primary cells (male) were purchased from PromoCell (Germany).<br>Huh7 was provided by Dr. Reinhild Prange, MCF7 by Dr. Walburgis Brenner, HCT116 by Dr. Thomas Kindler, (all University Medical Center, Mainz, Germany).
Authentication	All cell lines were identified via STR profiling performed by Microsynth, Germany and Eurofins, Germany. Normal Human Epidermal Keratinocytes (NHEK) were purchased from PromoCell.
Mycoplasma contamination	Mycoplasma contamination was excluded through PCR-based mycoplasma assay performed by Microsynth, Germany and Eurofins, Germany.
Commonly misidentified lines (See ICLAC register)	No commonly misidentified line was used in this study.

Plants

Seed stocks	No plants were used in this study.
Novel plant genotypes	No plants were used in this study.
Authentication	No plants were used in this study.

Flow Cytometry

Plots

Confirm that:

☒ The axis labels state the marker and fluorochrome used (e.g. CD4-FITC).

☒ The axis scales are clearly visible. Include numbers along axes only for bottom left plot of group (a 'group' is an analysis of identical markers).

☒ All plots are contour plots with outliers or pseudocolor plots.

☒ A numerical value for number of cells or percentage (with statistics) is provided.

Methodology

Sample preparation	Cells were transfected as described in detail in the manuscript. This includes cell seeding, transfection and harvest.
Instrument	Beckman Coulter, flow cytometer FC500
Software	Beckman Coulter, CXP analysis software
Cell population abundance	Viable GFP positive cells were sorted by using FSC, SSC and GFP gating. Thereby, approximately 10% of the transfected cells displaying high GFP signal were present in post sort fractions.
Gating strategy	Boundries between negative and positive populations were determined using untreated or control treated cells.

☒ Tick this box to confirm that a figure exemplifying the gating strategy is provided in the Supplementary Information.
